# Supplementary figures and images for: Lipobiotin-capture magnetic bead assay for isolation, enrichment and detection of Mycobacterium tuberculosis from saliva
Source: PLoS One. 2022 Jul 15;17(7):e0265554. doi: 10.1371/journal.pone.0265554 (PMC9286268; doi:10.1371/journal.pone.0265554)

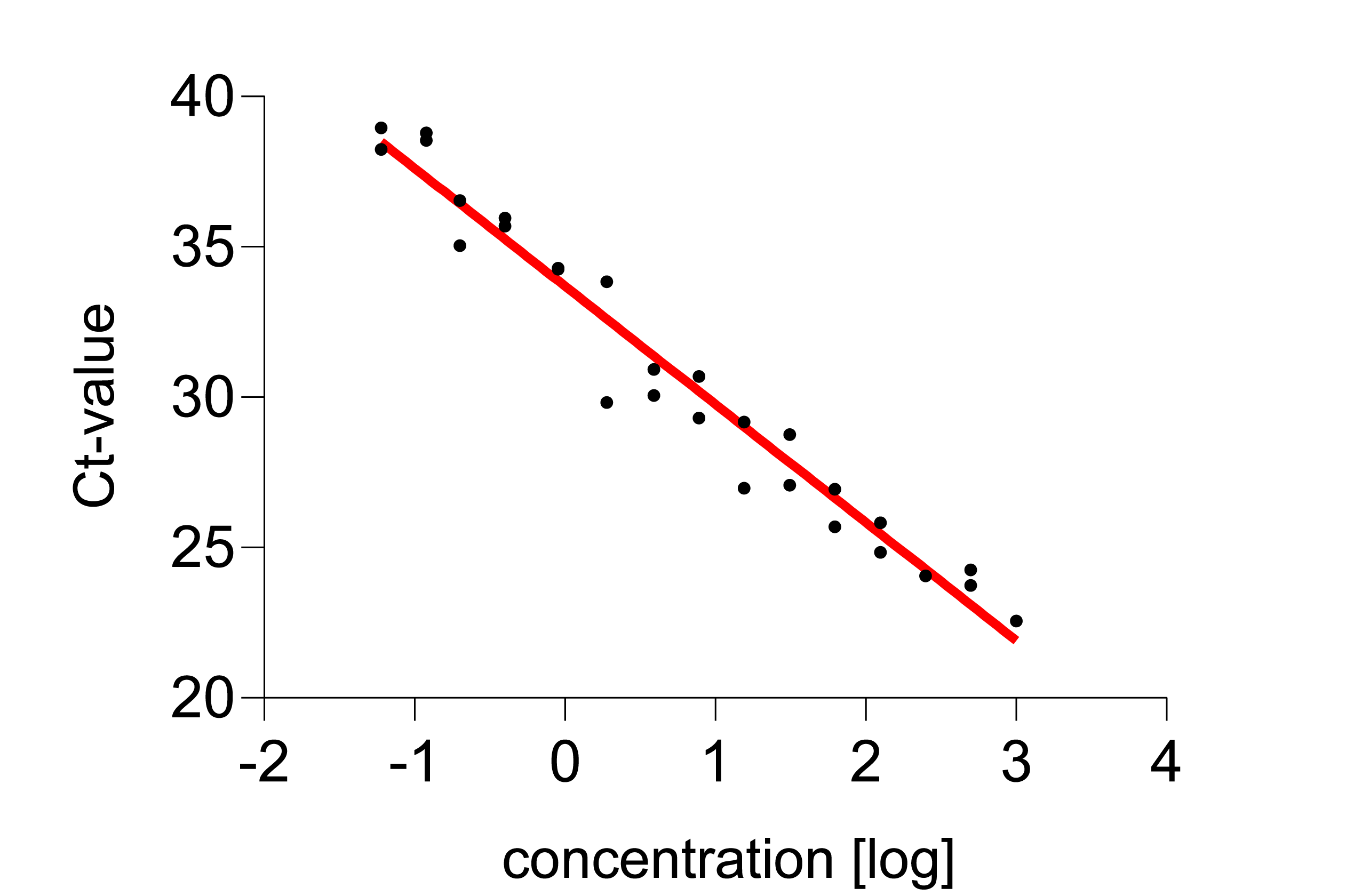

Supplement: S1 Fig — After DNA extraction, two-fold serial dilutions were analysed by qPCR detecting a region in Rv2819 from Mtb. A simple linear regression was conducted, revealing the efficiency of this qPCR reaction (E = 1.77, 77%). Using this standard curve, the (relative) DNA concentration of unknown samples (based on their ct-values) was calculated based on the formula Y(Ct) = -3,998*X(log(concentration) + 33,56. Obtained relative DNA concentrations were used to estimate the fold enrichment of Mtb DNA in LMB assay treated samples as given in the main text. (TIF) [file pone.0265554.s001.tif]

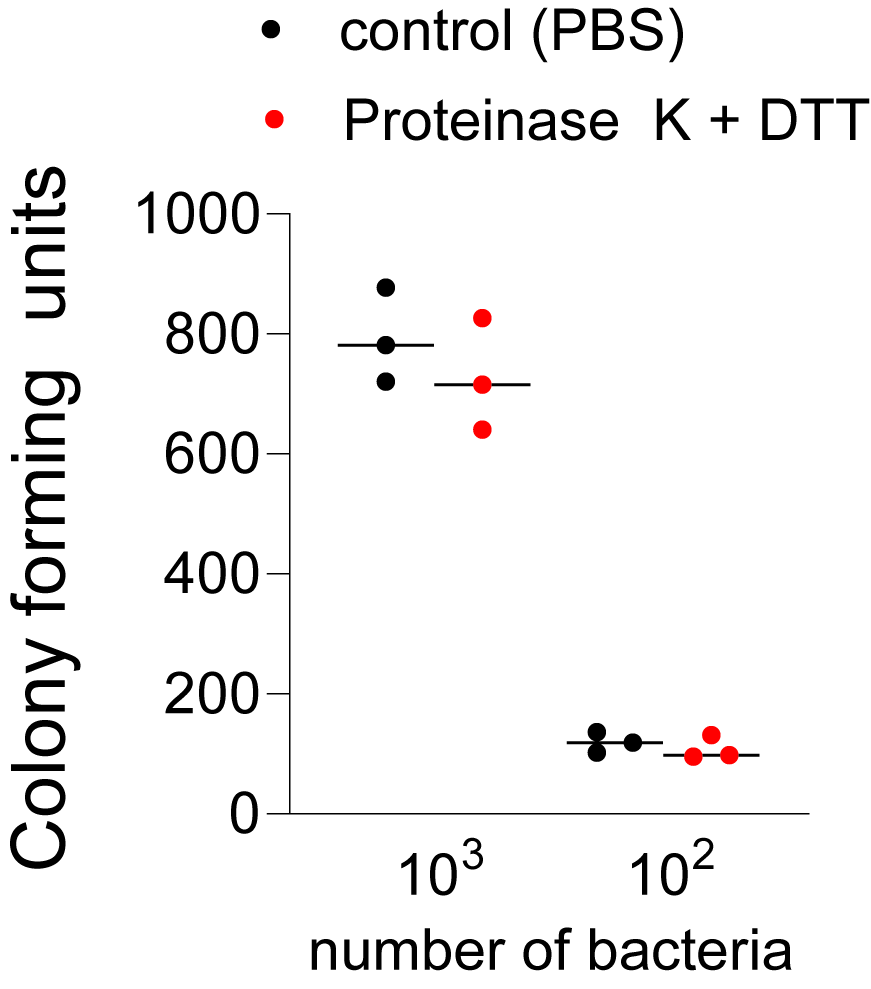

Supplement: S2 Fig — The indicated concentration of Mtb H37Rv was incubated in the absence and presence of Proteinase K (18U/ml) plus DTT (2mM) for 1h at 37°C. Colony forming units were determined as described in Material and Methods. Statistical analysis using an U-Test comparing control (PBS) and Proteinase K+DTT treated samples showed no significant differences between the groups (p = 0.3753). Line at each condition indicates the median of 3 independent experiments. (TIF) [file pone.0265554.s002.tif]

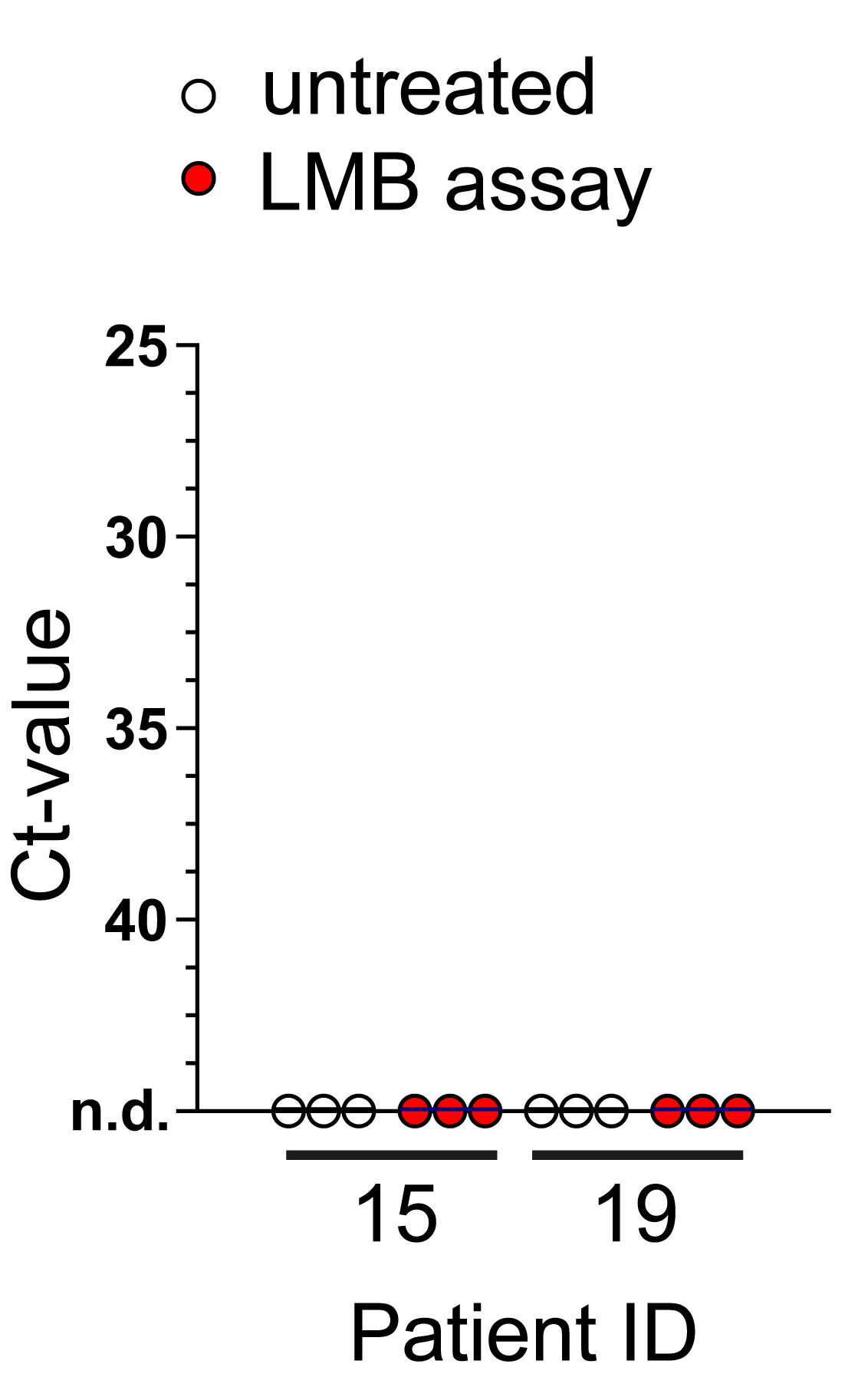

Supplement: S3 Fig — From two healthy subjects (sample ID 15 and 19), which were tested positive in the first experiment (compare to Fig 5) and that were available for re-testing, saliva was collected. Subsequently, sample was split into two fractions and left untreated or incubated with LMBs (LMB assay) before undergoing heat treatment, DNA isolation, and qPCR analysis. Analysis of three technical replicates is shown with the line at median. n.d., not detectable. (TIF) [file pone.0265554.s003.tif]
